# Supplementary material for: Effects of blast exposure on anxiety and symptoms of post-traumatic stress disorder (PTSD) among displaced Ukrainian populations
Source: PLOS Glob Public Health. 2024 Apr 11;4(4):e0002623. doi: 10.1371/journal.pgph.0002623 (PMC11008772; doi:10.1371/journal.pgph.0002623)
Supplement: S2 Table — (DOCX) [file pgph.0002623.s004.docx]

| S2 Table. OLS regression models showing standardized associations between **generalized anxiety** and independent variables | **Model 1** | **Model 2** | **Model 3** | **Model 4** | **Model 5a** | **Model 5b** |
| --- | --- | --- | --- | --- | --- | --- |
| Blast exposure | 0.09*** | 0.08*** | 0.08*** | 0.07*** | 0.06** | 0.07** |
| Pre-existing anxiety or depression | 0.15*** | 0.15*** | 0.14*** | 0.14*** | 0.14*** | 0.13*** |
| **Demographics** |  |  |  |  |  |  |
| IDPs (ref. = refugees) | 0.10*** | 0.08*** | 0.08*** | 0.06*** | 0.07*** | 0.06*** |
| Female (ref. = male) | 0.16*** | 0.16*** | 0.16*** | 0.16*** | 0.16*** | 0.16*** |
| Age group (ref. = 35-44) |  |  |  |  |  |  |
| 18-24 | 0.01 | 0.01 | 0.02 | 0.02* | 0.02* | 0.02* |
| 25-34 | -0.01 | -0.01 | -0.00 | 0.00 | 0.00 | 0.00 |
| 45-54 | 0.00 | 0.00 | -0.00 | 0.00 | 0.00 | 0.00 |
| 55-64 | 0.00 | 0.01 | 0.01 | 0.01 | 0.01 | 0.01 |
| 65+ | 0.00 | 0.01 | 0.01 | 0.01 | 0.01 | 0.01 |
| Language at home (ref. = Ukrainian) |  |  |  |  |  |  |
| Russian | -0.02 | -0.02 | -0.02 | -0.01 | -0.01 | -0.01 |
| Other | 0.00 | 0.01 | 0.01 | 0.00 | 0.00 | 0.00 |
| Education (ref. = high) | 0.00 | 0.01 | 0.01 | -0.01 | -0.01 | -0.01 |
| Origin (ref. = Kiev) |  |  |  |  |  |  |
| North | -0.02 | -0.02 | -0.02 | -0.02* | -0.02* | -0.02* |
| East | 0.03* | 0.04** | 0.03* | 0.02 | 0.02 | 0.02 |
| South | 0.04*** | 0.04*** | 0.04*** | 0.03** | 0.03** | 0.03** |
| West | 0.02 | 0.02 | 0.02 | 0.01 | 0.02 | 0.01 |
| Central | -0.00 | -0.00 | -0.00 | -0.01 | -0.01 | -0.01 |
| Survey completion month by participants (ref. = April) |  |  |  |  |  |  |
| May | -0.06*** | -0.06*** | -0.06*** | -0.06*** | -0.06*** | -0.06*** |
| June | -0.03* | -0.02 | -0.03* | -0.03* | -0.03* | -0.03* |
| July | -0.03** | -0.03** | -0.03** | -0.03* | -0.03* | -0.03* |
| **Family/Network** |  |  |  |  |  |  |
| Marital status (ref. = married/cohab.) |  |  |  |  |  |  |
| Unmarried |  | -0.00 | -0.00 | -0.01 | -0.01 | -0.01 |
| Separated/divorced/widowed |  | -0.02 | -0.02 | -0.03** | -0.03** | -0.03** |
| Care responsibilities for over 18s |  | 0.06*** | 0.05*** | 0.05*** | 0.05*** | 0.05*** |
| Left anyone from immediate family |  | 0.02 | 0.02 | 0.02 | 0.01 | 0.01 |
| **Living circumstances** |  |  |  |  |  |  |
| Chronic disease |  |  | 0.03* | 0.02 | 0.02 | 0.02 |
| Accommodation size = small (ref. = just right or too big) |  |  | 0.07*** | 0.05*** | 0.05*** | 0.05*** |
| **Access to resources** |  |  |  |  |  |  |
| Food |  |  |  | -0.15*** | -0.15*** | -0.15*** |
| Governmental welfare payments |  |  |  | -0.02 | -0.02 | -0.02 |
| Healthcare access |  |  |  | -0.05*** | -0.05*** | -0.05*** |
| **Interactions** |  |  |  |  |  |  |
| Blast exposure × Displacement type |  |  |  |  | 0.02 |  |
| Blast exposure × Mental health |  |  |  |  |  | 0.01 |
|  |  |  |  |  |  |  |
| Constant | 2.37*** | 2.24*** | 2.12*** | 3.62*** | 3.61*** | 3.63*** |
| *Adj. R²* | 0.073 | 0.077 | 0.083 | 0.107 | 0.108 | 0.107 |
| *F* | 32.23*** | 28.24*** | 28.28*** | 33.81*** | 32.70*** | 32.68*** |

*: *p* <.05.

**: *p* <0.01.

***: *p* <.001.
