# Supplementary material for: Effects of blast exposure on anxiety and symptoms of post-traumatic stress disorder (PTSD) among displaced Ukrainian populations
Source: PLOS Glob Public Health. 2024 Apr 11;4(4):e0002623. doi: 10.1371/journal.pgph.0002623 (PMC11008772; doi:10.1371/journal.pgph.0002623)
Supplement: S3 Table — (DOCX) [file pgph.0002623.s005.docx]

| S3 Table. OLS regression models showing standardized associations between **nightmares** and independent variables | **Model 1** | **Model 2** | **Model 3** | **Model 4** | **Model 5a** | **Model 5b** |
| --- | --- | --- | --- | --- | --- | --- |
| Blast exposure | 0.12*** | 0.12*** | 0.12*** | 0.10*** | 0.10*** | 0.10*** |
| Pre-existing mental health | 0.13*** | 0.12*** | 0.12*** | 0.11*** | 0.11*** | 0.10*** |
| **Demographics** |  |  |  |  |  |  |
| IDPs (ref. = refugees) | 0.06*** | 0.06*** | 0.05*** | 0.04** | 0.04 | 0.08** |
| Female (ref. = male) | 0.08*** | 0.08*** | 0.08*** | 0.08*** | 0.08*** | 0.08*** |
| Age group (ref. = 35-44) |  |  |  |  |  |  |
| 18-24 | -0.02 | -0.02 | -0.01 | -0.01 | -0.01 | -0.01 |
| 25-34 | 0.01 | 0.01 | 0.02 | 0.02* | 0.02* | 0.02* |
| 45-54 | -0.04** | -0.04** | -0.04*** | -0.04*** | -0.04*** | -0.04*** |
| 55-64 | -0.07*** | -0.07*** | -0.07*** | -0.07*** | -0.07*** | -0.07*** |
| 65+ | -0.03** | -0.03** | -0.03** | -0.03** | -0.03** | -0.03** |
| Language at home (ref. = Ukrainian) |  |  |  |  |  |  |
| Russian | -0.05*** | -0.05*** | -0.05*** | -0.04*** | -0.04*** | -0.04*** |
| Other | -0.02 | -0.02 | -0.02 | -0.02 | -0.02 | -0.02 |
| Education (ref. = high) | 0.04*** | 0.04*** | 0.04*** | 0.03* | 0.03* | 0.03* |
| Origin (ref. = Kiev) |  |  |  |  |  |  |
| North | 0.03* | 0.03* | 0.03* | 0.02 | 0.02 | 0.02 |
| East | 0.02 | 0.02 | 0.02 | 0.01 | 0.01 | 0.01 |
| South | 0.04** | 0.04** | 0.04** | 0.03* | 0.03* | 0.03* |
| West | 0.01 | 0.01 | 0.01 | 0.00 | 0.00 | 0.00 |
| Central | -0.00 | -0.00 | -0.00 | -0.01 | -0.01 | -0.01 |
| Survey completion month by participants (ref. = April) |  |  |  |  |  |  |
| May | -0.02 | -0.02 | -0.02 | -0.02 | -0.02 | -0.02 |
| June | -0.01 | -0.01 | -0.01 | -0.01 | -0.01 | -0.01 |
| July | -0.02 | -0.02 | -0.02 | -0.01 | -0.01 | -0.01 |
| Rural origin type (ref. = urban) | 0.02 | 0.02 | 0.02 | 0.01 | 0.01 | 0.01 |
| **Family/Network** |  |  |  |  |  |  |
| Marital status (ref. = married/cohab.) |  |  |  |  |  |  |
| Unmarried |  | -0.00 | -0.01 | -0.01 | -0.01 | -0.01 |
| Separated/divorced/widowed |  | 0.00 | 0.00 | -0.01 | -0.01 | -0.01 |
| Care responsibilities for over 18s |  | 0.04*** | 0.04** | 0.03** | 0.03** | 0.03** |
| Left anyone from immediate family |  | 0.02* | 0.02* | 0.02 | 0.02 | 0.02 |
| **Living circumstances** |  |  |  |  |  |  |
| Chronic disease |  |  | 0.05*** | 0.04*** | 0.04*** | 0.04*** |
| Accommodation size = small (ref. = just right or too big) |  |  | 0.04*** | 0.01 | 0.01 | 0.01 |
| **Access to resources** |  |  |  |  |  |  |
| Food |  |  |  | -0.13*** | -0.13*** | -0.13*** |
| Governmental welfare payments |  |  |  | -0.02 | -0.02 | -0.02 |
| Healthcare access |  |  |  | -0.05*** | -0.05*** | -0.05*** |
| **Interactions** |  |  |  |  |  |  |
| Blast exposure × Displacement type |  |  |  |  | -0.00 |  |
| Blast exposure × Mental health |  |  |  |  |  | -0.01 |
|  |  |  |  |  |  |  |
| Constant | 1.67*** | 1.58*** | 1.51*** | 2.24*** | 2.24*** | 2.24*** |
| *Adj. R²* | 0.057 | 0.059 | 0.062 | 0.082 | 0.082 | 0.082 |
| *F* | 23.46*** | 20.36*** | 20.00*** | 24.30*** | 23.56*** | 23.57*** |

*: *p* <.05.

**: *p* <0.01.

***: *p* <.001.
