# Supplementary material for: Effects of blast exposure on anxiety and symptoms of post-traumatic stress disorder (PTSD) among displaced Ukrainian populations
Source: PLOS Glob Public Health. 2024 Apr 11;4(4):e0002623. doi: 10.1371/journal.pgph.0002623 (PMC11008772; doi:10.1371/journal.pgph.0002623)
Supplement: Attachment — Submitted filename: Response to Reviewers.docx [file pgph.0002623.s006.docx]

October 20 2023

*PLoS Global Public Health*

Dear reviewers,

Please accept our sincerest gratitude for your time and effort in providing thoughtful feedback on our manuscript. Your responses has helped us in transforming this manuscript for the better – and we gratefully appreciate it.

Please find the following comments in blue to your kind suggestions.

Warm wishes,

Dr. Ken Brackstone, Ph.D.

University of Southampton

Clinical Informatics Research Unit,

Care of the National Blood and Transplant Service, MP 852,

Coxford Road,

Southampton, UK

SO16 5AF

Journal Requirements:

1. Please provide additional information regarding the considerations made for the refugees included in this study. For instance, please discuss whether participants were able to opt out of the study and whether individuals who did not participate receive the same treatment offered to participants.

Thanks – no problem. We now state in the ethical approval section that individuals who did not participate received the same survey information as those who did, and that participants could opt out of the study at any time during survey completion.

2. Our staff editors have determined that your manuscript is likely within the scope of our Global Mental Health: challenges, opportunities, and the future of the field. This editorial initiative is headed by a team of Guest Editors for PLOS GPH: Rochelle Burgess (University College of London) and Dixon Chibanda (University of Zimbabwe and London School of Tropical Medicine and Hygiene). The Collection invites researchers to submit original research which engages with, or disrupts, the urgent needs across the global mental health landscape.

Thank you. Whilst we can see that the manuscript may be suitable for the Global Mental Health editorial, we would appreciate if you could remove this manuscript from consideration there. Respectfully, we would gratefully prefer to publish it in PLOS Global Public Health instead. Thank you very much for bringing this to our attention.

3. Please amend your detailed Financial Disclosure statement. This is published with the article. It must therefore be completed in full sentences and contain the exact wording you wish to be published.

a. State the initials, alongside each funding source, of each author to receive each grant.
b. State what role the funders took in the study. If the funders had no role in your study, please state: “The funders had no role in study design, data collection and analysis, decision to publish, or preparation of the manuscript.”
c. If any authors received a salary from any of your funders, please state which authors and which funders.

Thanks for pointing this out. We have now reworded the Financial Disclosure statement on the resubmission screen. I hope that this sounds better: **This research was funded by Public Policy@Southampton (MH, KB, BPH), the Clinical Informatics Research Unit, University of Southampton (KB, MH), and the ESRC Centre for Population Change (BPH). The funders had no role in study design, data collection and analysis, decision to publish, or preparation of this manuscript.**

4. Please provide separate figure files in .tif or .eps format only and remove any figures embedded in your manuscript file. Please also ensure all files are under our size limit of 10MB.

Thank you. The figures have now been uploaded as TIF files. Please reach out if you need us to submit them as something else instead.

**Reviewer #1**

• Please refer to the following finding in the Introduction as part of the literature review: Prevalence of depression, anxiety and post-traumatic stress in war- and conflict-afflicted areas: A meta-analysis. Front Psychiatry. 2022 Sep 16; 13: 978703. doi: 10.3389/fpsyt.2022.978703.

We appreciate your kind suggestions in making this a stronger a manuscript. I can confirm that I have now included your suggestions throughout our manuscript. This article has been included in our Introduction section to provide extra context for mental health symptoms experienced by civilians in war and conflicted areas. Thank you.

• The authors can discuss the finding of the following paper and how their findings are similar or different.

Depression, anxiety and post-traumatic stress during the 2022 Russo-Ukrainian war, a comparison between populations in Poland, Ukraine, and Taiwan. Sci Rep. 2023 Mar 3; 13(1):3602. doi: 10.1038/s41598-023-28729-3.

Your second suggested article helps us to strengthen the implications section, which demonstrates how Russia’s invasion of Ukraine has resulted in a variety of mental health outcomes pertaining to PTSD. Thank you.

• Under discussion, please recommend the use of Internet or digit psychotherapy to help Ukrainians:

Search PubMed for: This meta-analysis provides strong support for the effectiveness of dCBT-I in treating insomnia. dCBT-I has potential to revolutionise the delivery of CBT-I, improving the accessibility and availability of CBT-I content for insomnia patients worldwide.

Your third suggestion fitted nicely in the implications section, too, which now suggests that digital CBT in general could be one option offered to all Ukrainians due to its excellent accessibility.

Thank you very much!

**Reviewer #2:**

• Use third person and not first or second person in sentence construction – see line 32, 33, 35, 138, 163

Many thanks for your kind comments on this manuscript – they are gratefully appreciated. Thank you for spotting these language discrepancies. I can confirm that there are now no instances of first or second person throughout the manuscript.

• Suggest title be improved as: Effects of Blast exposure on Post-Traumatic Stress Disorder (PTSD) among displaced Ukrainian population

We heartily agree that the title needed to be improved and less wordy. I have taken your advice and renamed it to your suggestion above, although I have also included ‘anxiety’ in the title, too. It is now: **Effects of Blast Exposure on Anxiety and Symptoms of Post-Traumatic Stress Disorder (PTSD) among Displaced Ukrainian Populations**

**Abstract**

• Structure it so that the following subsections are visible: Background, Aim, Methods, Result and conclusion and then merged in one as opposed to sub-sections

Many thanks. I tried to make this work, although the having subsections in the abstract does not seem to fit the requirements or style for PLoS Global Public Health. I tried to find a few published studies that had visible subsections, but I could not find any. Thus, I have proceeded without subsections in this instance.

• Abstract is quite big

Agreed! It was 372 words before. It is now 283 words. Many thanks for pointing this out.

• First sentence in abstract and Introduction should address the problem investigated, which is related to mental illnesses and PTSD before addressing the cause – in this case, blast exposure.

The abstract/introduction sections have now been completely reshuffled and reworded. In both sections, we have now written: **Anxiety and post-traumatic stress disorder (PTSD) are common among war-affected communities, especially among those forcibly displaced during war [1, 2]. Blast exposure may be one important contributor of such symptoms.**

We agree with you that it is better to set the scene with the problem (i.e., anxiety and PTSD) before going into the potential cause of the problem. Thank you for this tip.

**Introduction**

• Aims: Line 138-147- Aims need to be shorted and straight forward and therefore need to be re-written

Many thanks for this suggestion – I agree. The aims have been rewritten and are now less wordy than before. The 3 aims are now much more specific. We have also added a hypothesis to the final aim relating to blast exposure and the moderation models.

**Methods**

• Line 148: Subtitle should be “Methodology”

Thanks. This has now been updated.

• Methods are not flowing and please structure it as below:

o Study design
o Study setting
o Study population
o Sample size
o Selection criteria (Inclusion and exclusion)
o Tools used
o Sampling procedures
o Procedures for data collection
o Data management and quality control
o Statistical data analysis
o Ethical consideration

Thank you. The results section now contains most of these subheadings. These include: study design and setting; study population; sample size; measures; quality controls; statistical analysis; and ethical approvals. Selection criteria was irrelevant to this study. These sections/items were included as per the STROBE checklist for cross-sectional studies, which I have attached to this resubmission.

**Results**

• Results to be sub-sectioned and given clear sub-titles

The Results section now has extra sub-sections throughout. Each set of analyses now has its own sub-section: demographic characteristics, blast exposure and mental health, associations with flashback frequency, associations with generalized anxiety, and associations with nightmares.

• Sub-section should be “Demographic characteristics”

Thanks – agreed. A sub-section has been added and is labelled demographic characteristics.

• Line 239 -Table 1: Demographics of Internally Displaced People within Ukraine and Refugees, Percentages should in the sub-title at the top of the cell in each column and not on every figure and % should come first and then (n) i.e. % (n).

Thanks – agreed. The tables have now been updated to reflect this. % (n) is now the subheading for every column. Percentages now come first, followed by the number of participants in brackets. I have removed the % symbol for cell, which previously took up much space and looked untidy in the tables.

• The % seems not to add up since they are calculated on each item. Suggest that it should be calculated based on the total i.e. Total Gender is n=8326 (100%) for both IDP and Refugees. What is the % contribution of male and female for IDP and refugees based on the total number such that when added up they give 100%. This should be done all through the table.

Thanks for spotting these important errors. There were 7 variables/instances where they did not equal 100% in Table 1.

• Line 254 -Table 2: Outcome Percentages of Internally Displaced People within Ukraine and Refugees Categorical Outcomes (M ± SD) ----- See table 1 above. % should not be on every figure. Let it be put on the sub-title in each column

As per Table 1 above, the tables have now been updated to reflect this. % (n) is now the subheading for every column. Percentages now come first, followed by the number of participants in brackets. I have removed the % symbol for cell, which previously took up much space and looked untidy in the tables.

• And % should add up as commented in Table 1 above

Thanks for spotting this. All variables in Table 2 now add up to 100%.

• Line 258 - Table 3: Outcome Percentages of Internally Displaced People within Ukraine and Refugees Continuous Outcomes (M ± SD) – Are the figure reported % or something else? What are the units of those figures?

Thanks for this. Table 3 was previously given the wrong title. These are, in fact, means and standard deviations. I have now updated the title, which now reads: **Table 3. Means and Standard Deviations of Continuous Outcomes Comparing Internally Displaced People and Refugees.**

• Line 274- Table 4: OLS regression models showing associations between flashback frequency and independent variables – Table needs a key. What is ****? What does – and + values in the table mean in the regression analysis?

Great – thank you. The key for Table 4 has now been added. We decided to remove the ± values (which originally meant marginally significant) from the regression table.

• Line 319- Table 5. OLS regression models showing associations between generalized anxiety and independent variables -- Table needs a key. What is ****? What does – and + values in the table mean in the regression analysis?

Great – thank you. The key for Table 5 has now been added. We decided to remove the ± values (which originally meant marginally significant) from the regression table.

• Line 359 - Table 6. OLS regression models showing associations between nightmares and independent variables --- Table needs a key. What is ****? What does – and + values in the table mean in the regression analysis?

Great – thank you. The key for Table 6 has now been added. We decided to remove the ± values (which originally meant marginally significant) from the regression table.

**Discussion**

• I suggest that Discussion be sub-sectioned according to the results.

Many thanks for this suggestion. The first paragraph of the Discussion starts off with a general high-level summary of the key findings, but then follows into each of the sub-sections of the results as per your recommendation. Before, the results were slightly all over the place. They do not contain sub-headings since we were keen to follow a similar style to articles published in PLoS Global Public Health, but it should now be clearer to the reader that it follows the same trail as the results.

• Use third person and not first or second person in sentence construction

Thanks. All instances of second and third person have been deleted throughout the Discussion section.

• What is the role of Blasts exposure to Mental illnesses among the new cases and those with existing cases?

Good point. We clarified one of the paragraphs which touches on this, especially we did not assess previous/existing instances of PTSD. This has now been touched upon.

**“Although we did not measure previous PTSD symptoms, some participants in this study may have already experienced trauma after having been displaced during the 2014 separatist conflict in the eastern regions of Ukraine [33]. Around 1.5 million people were forced to leave their homes as a result of this conflict, and many settled close to the border of the separatist regions, which were and are currently war zones across 2022-23 [34]. Studies have shown that between 21% of the original Ukrainian IDP population met criteria for PTSD or Complex PTSD (CPTSD) [35]. If they fled for the second time in their lives, it is likely that their PTSD symptoms most likely would have become even more acute due to blast exposure.”**

**Conclusion**

• Use third person and not first or second person in sentence construction

Thanks. All instances of third person have been deleted throughout the conclusion.

• Conclusion should be made on each of the key findings on each sub section

Thank you. The Conclusion section is now much more concise than before. Each sentence summarises each key finding in the Discussion section.

**Reviewer #3**:

• Please make the title smaller

Many thanks – agreed that it was far too long before. It is now: **Effects of Blast Exposure on Anxiety and Symptoms of Post-Traumatic Stress Disorder (PTSD) among Displaced Ukrainian Populations**

• Break the abstract in separate parts like introduction/background, methods, results, conclusion.

Many thanks. I tried to make this work, although having subsections in the abstract does not seem to fit the requirements or style for PLoS Global Public Health. I tried to find a few published studies that had visible subsections, but I could not find any. Thus, I have proceeded without subsections in this instance.

• Please improve the justification of the study. Add why this study is needed in the context.

The introduction has been drastically reshaped to answer these important questions. For example, we have pointed out on many occasions why it is important to assess blast exposure as one possible associate with PTSD. And we have strongly claimed, after the aims and hypotheses, that the study fills a crucial evidence gap that has not been explored in the context of Russia’s invasion of Ukraine and the subsequent mental health outcomes of IDPs and refugees.

• I cannot find any tool in the method that were used to assess the PTSD. If the PTSD is not assessed, then how author say PTSD in the title. I think should be careful about their study planning and reporting.

Thank you. I agree that it was quite unclear before. We have now included a **PTSD symptoms** section under the Measures to discuss how we assessed flashbacks and nightmares (single items), including their response options and corresponding means and standard deviations.

• Follow the reporting guidelines STOBE and cover all the sections in the STOBE.

- Give description of the population	- Add a section for the questionnaire development. - Add a section for the data collection techniques. - Add a section for the bias. - Add a section for the dependent variables and independent variables. - Add the exclusion and inclusion criteria. - Report the sampling techniques. - Report the sample size calculation. - Report the validity and reliability of the tools used in this study. - Cite the literature from where author used the tools	- Provide equitable description and the scoring of the tools

Thank you for this. The results section now contains most of these subheadings. These include: study design and setting; study population; sample size; measures; quality controls; statistical analysis; and ethical approvals. Selection criteria was irrelevant to this study. The relevant tools used have been cited from the literature, including their descriptions and scorings of the tools. Validity and reliability (i.e., correlations between items in the 2 item scale) have been used where appropriate, although reliability was more difficult with single item measures. Although, these limitations have addressed in the Discussion section. All your suggestions have been touched upon in the **STROBE checklist for cross-sectional studies**, which I have attached to this resubmission.

- Discussion should be more contractive. Add sections:
  • Limitations of the study
  • Future research	• Recommendation for the stakeholders
  • What is new in this study?

Many thanks for this suggestion. The Discussion now contains additional sections, which we did not include before. These include the **Implications** section, which contains recommendations for the stakeholders and what the study delivers that is new; and a **Limitations and future research** section. We have combined limitations and future directions since they tend to link to each other; the limitations inform the future research. This also mirrors other studies recently published in PLoS Global Public Health.

**Reviewer #4:**

• “First, chi-square (x²) analyses were used to examine associations between displacement type and demographics and outcome predictors”. Give all rationale of the test.• Please specify when the tests were done.

Great – thanks. We now explain slightly better why we used *x²* tests, since they relate to the descriptive statistics in Tables 1-2. **“Descriptive statistics and chi-square (*x²*) analyses were first conducted to compare demographic characteristics among IDPs and refugees, and then to compare incidence of blast exposure and other categorical variables.”** This should also provide a better idea of the order in which the tests were administered. In fact, the entire statistical analysis section has been bolstered, and the order in which the tests were completed should hopefully now be easier to follow. Thanks again for this.

• All the tests should be consistent with the study objective.

The study aims in the introduction section has been re-written, so it should hopefully be clearer as to why we are running these specific analyses/tests, and how they link to each of our three aims.

• How were the variables selected in the adjusted model, please meet the selection criteria?
Did the assumptions of the model fitted, report the all the assumptions of the model? Did author check for confounding, Effect modification, and multi-collinearity?

Many thanks for this. Your insight has strengthened our argument for running the regression models the way we have. Indeed, we tested key assumptions beforehand, but we neglected to say so in the statistical analysis section.

We now state that each model consisted of factors/IVs that with P values equal to, or smaller than, 0.2 in univariate analyses which were conducted beforehand. Thus, each model varies slightly according to what was significant in the univariate analysis. For example, **not receiving governmental welfare payments** and **leaving behind somebody in one’s close family after displacement** were significantly associated with generalized anxiety in the univariate analyses (and were included in the model predicting generalized anxiety), but these variables were nowhere near significantly associated with flashback symptoms (and thus, we did not include them in the adjusted model for flashbacks).

All assumptions, including tests for confounders, normality, and multicollinearity, were tested before and during model-building. Independent variables – apart from key demographic variables, which we included in all models as a default – were added according to the support of the literature. The now-bolstered introduction section now touches on a few of these variables included in our regression models.

Thank you so much again for this.

• As the author used linear regression, were the assumptions met, importantly normality, and multicollinearity? The author cannot fit the model (putting all variables in the model) without following the model-building criteria.

Thank you for your comments. As above, we tested key assumptions beforehand, but we neglected to say so in the statistical analysis section. All assumptions, including tests for confounders, normality, and multicollinearity, were tested before and during model-building.

• After fitting the model, did they check the model's fitness? There are many ways to check the model fitness, and predictive accuracies, which require providing the information. Submit the figures (for example Q-Q plots) of the model accuracies as supplementary files if required.

We did assess and report the fitness of each regression model. We opted for *R²*, which made the most sense to our reporting of each model, which we acknowledge contains a wide range of predictors and interactions. These are reported at the bottom of each OLS regression table (Tables 4-6) under each column as the models build up. We now state in the statistical analyses section that **“all models were described in terms of coefficients of determination (*R²*), which are indicative of how well the fitted models predicted the key outcome variables”.**

• Require reporting the post-estimation test VIF.

Thanks – good point. VIFs for all regression models ranged between 1 and 2, suggesting that the models thankfully did not contain multicollinearity issues.

We now state in the statistical analysis section: **“All assumptions, including tests for confounders, normality, and multicollinearity (VIFs), were tested for during model-building. Thus, any variables that we report passed the assumption checks.”** This should reassure readers that all assumptions checks were satisfactory for the models.

• Research question requires outlines in the justification and the model should be built to address these questions

Thanks again. As above, the study aims in the introduction section has been re-written and simplified. It should now be clearer as to why we are running these specific tests/analyses, and how they link to each of our three aims.

Thank you so much for your kind comments.
