# Supplementary material for: Effects of blast exposure on anxiety and symptoms of post-traumatic stress disorder (PTSD) among displaced Ukrainian populations
Source: PLOS Glob Public Health. 2024 Apr 11;4(4):e0002623. doi: 10.1371/journal.pgph.0002623 (PMC11008772; doi:10.1371/journal.pgph.0002623)
Supplement: Attachment — Submitted filename: Response to Reviewers.docx [file pgph.0002623.s007.docx]

December 18 2023

*PLoS Global Public Health*

Dear reviewers,

Please, once again, accept our sincerest gratitude for your time and effort in providing thoughtful feedback on our manuscript. Your responses has helped us in transforming this manuscript for the better – and we gratefully appreciate it.

Please find the following comments in blue to your kind suggestions.

Warm wishes,

Dr. Ken Brackstone, Ph.D.

University of Southampton

Clinical Informatics Research Unit,

Care of the National Blood and Transplant Service, MP 852,

Coxford Road,

Southampton, UK

SO16 5AF

Journal Requirements:

1. Please update your online Competing Interests statement. If you have no competing interests to declare, please state: “The authors have declared that no competing interests exist.”

Thanks. This has been updated in the manuscript and the journal submission system.

2. 2. Please review your reference list to ensure that it is complete and correct. If you have cited papers that have been retracted, please include the rationale for doing so in the manuscript text, or remove these references and replace them with relevant current references. Any changes to the reference list should be mentioned in the rebuttal letter that accompanies your revised manuscript. If you need to cite a retracted article, indicate the article’s retracted status in the References list and also include a citation and full reference for the retraction notice.

Thank you. As far as we can tell, all references are correct and complete.

**Reviewer #2**

• Line 139: Use past tense

Good spot – thank you. There was also a tense issue in the abstract, which I have also corrected.

• Study design and setting: Study setting is not included

Thanks – good point. There is no study ‘setting’ since this was an online survey, so I have deleted ‘study setting’ from the sub-title.

• Line 158: There should be “Study data collection procedures” before you go to Measures

Thanks. Following on from the comment above, I have combined ‘Study design’ and ‘procedure’ together to form a new sub-title: “Study design and procedure” as per journal guidelines. This makes better sense, especially since this paragraph already describes the study design and procedure in one section. Thank you for pointing this out and for helping to clarify these sections.

• In tables: < .001, write it as “p<0.001

Great – thanks. All done. The p values now state p < 0.001 throughout the table columns.

• Line 263: write < .001 as “p<0.001

Thanks for this. All done.

Thank you very much!
